# Effects of Magnesium Imbalance on Root Growth and Nutrient Absorption in Different Genotypes of Vegetable Crops

**DOI:** 10.3390/plants12203518

**Published:** 2023-10-10

**Authors:** Shuai Qu, Huixia Li, Xueke Zhang, Jingbo Gao, Rui Ma, Ling Ma, Jing Ma

**Affiliations:** 1 College of Agriculture, Ningxia University, Yinchuan 750021, China; qustudy@163.com (S.Q.); mr41456966@163.com (R.M.); hearmaling045@163.com (L.M.); joyma1116@163.com (J.M.); 2 College of Civil and Hydraulic Engineering, Ningxia University, Yinchuan 750021, China; 13995100606@163.com; 3 College of Agronomy and Life Sciences, Shanxi Datong University, Datong 037009, China; jingbogao0927@163.com

**Keywords:** better Mg supply, tomato, cucumber, nutrient uptake, root growth, root exudation, organic acids

## Abstract

Magnesium (Mg) plays a crucial role in crop growth, but how Mg supply level affects root growth and nutrient absorption in vegetable crops with different genotypes has not been sufficiently investigated. In this study, the responses of tomato (*Solanum lycopersicum* L.) and cucumber (*Cucumis sativus* L.) crops to different levels of Mg supply were explored. Four levels of Mg treatment (i.e., 0.2, 1.0, 2.0, 3.0 mmol/L) were applied under hydroponic conditions, denoted as Mg0.2, Mg1, Mg2, and Mg3, respectively. The results showed that with increasing Mg levels, the plant biomass, root growth, and nutrient accumulation in both vegetable crops all increased until reaching their maximum values under the Mg2 treatment and then decreased. The total biomass per tomato plant of Mg2 treatment was 30.9%, 14.0%, and 14.0% higher than that of Mg0.2, Mg1, and Mg3 treatments, respectively, and greater increases were observed in cucumber plant biomass (by 54.3%, 17.4%, and 19.9%, respectively). Compared with the Mg0.2 treatment, the potassium (K) and calcium (Ca) contents in various plant parts of both crops remarkably decreased under the Mg3 treatment. This change was accompanied by prominently increased Mg contents in various plant parts and para-hydroxybenzoic acid and oxalic acid contents in root exudates. Irrespective of Mg level, plant biomass, root growth, nutrient accumulation, and root exudation of organic acids were all higher in tomato plants than in cucumber plants. Our findings indicate that excessive Mg supply promotes the roots to exude phenolic acids and hinders the plants from absorbing K and Ca in different genotypes of vegetable crops despite no effect on Mg absorption. A nutritional deficiency of Mg stimulates root exudation of organic acids and increases the types of exuded organic acids, which could facilitate plant adaption to Mg stress. In terms of root growth and nutrient absorption, tomato plants outperform cucumber plants under low and medium Mg levels, but the latter crop is more tolerant to Mg excess.

## 1. Introduction

Magnesium (Mg), which is an indispensable nutrient for crop growth and development, plays key roles in regulating enzyme activity, protein synthesis, lipid metabolism, and carbohydrate distribution [1,2]. Mg directly takes part in shoot photosynthesis and the production of photosynthetic metabolites. Therefore, Mg deficiency restricts crop growth and the distribution of photosynthetic products, causing severe declines in crop yield and quality. In the past five decades, Mg deficiency has become a widespread problem in agricultural production, leading to decreased Mg contents in grains, vegetables, and fruits [3]. Although research on plant Mg nutrition can be dated back to nearly 200 years ago, there have been insufficient studies elucidating the underlying mechanisms compared with other macronutrients and mesonutrients [4]. In contrast with nitrogen (N), phosphorus (P), and potassium (K) deficiencies, which strongly inhibit crop growth, Mg deficiency has less prominent adverse effects on crops, leading to the neglect of Mg fertilizer application in agricultural fields [5]. The consumption of food and vegetable crops is a major source of Mg for the human body. Given that many people in developed countries are deficient in Mg, poor human health caused by Mg deficiency is becoming increasingly serious [6,7], and deciphering the mechanisms of Mg nutrition in crops would be beneficial for human health.

In crops, Mg deficiency is attributable to absolute Mg deficiency and cation competition [8]. Absolute Mg deficiency is likely due to low soil Mg content as a consequence of excessive Mg loss, which usually occurs in acidic soils with a light texture and low cation exchange capacity [9]. Additionally, soil Mg can be depleted due to the non-recycling of crop residues and unbalanced inputs of mineral fertilizers under intensive cropping patterns [3]. The competition of Mg^2+^ with other cations, such as K^+^, calcium (Ca^2+^), ammonium (NH^4+^), and aluminum (Al^3+^), is also responsible for Mg deficiency in crops [9]. Another cause of Mg deficiency is the antagonistic interaction between Mg^2+^ and K^+^, which is the most common cation competition in plant mineral nutrition [10]. The problem of Mg deficiency can be solved through the field application of Mg fertilizer. However, when Mg fertilizer is applied in excess, nutrient stress affects plant photosynthetic and physiological properties, leading to retarded crop growth and development [11]. Moreover, Mg excess enhances cation antagonism and, as such, impairs K^+^ and Ca^2+^ absorption by crop plants. In particular, the application of K and Mg fertilizers is often insufficient or unbalanced in agricultural production. This results in an imbalance between K and Mg distributions in plants, which limits crop growth and nutritional quality [12]. Although Mg fertilizer regulation for plant nutrient absorption and Mg supply has been extensively investigated in various crops [10,13], few studies have looked at Mg nutrition in vegetable crops with different genotypes.

As plants take in and transport mineral nutrients depending on the roots, a decrease in the number of roots inevitably affects nutrient absorption and distribution, causing a decline in nutrient use efficiency [14]. In Mg-deficient plants, there are reduced plasmodesmata between phloem parenchyma cells in the main leaf veins, which hinders carbohydrate translocation from the roots to the shoots and consequently inhibits root growth [15]. The growth of crop roots relates closely to their exudation of organic acids. When plants are deficient in nutrients such as N, P, and iron (Fe), some physiological metabolic pathways are terminated, which allows for the accumulation of organic acids in large quantities and their subsequent release into the rhizosphere soil through the roots [16,17,18,19,20,21]. Low-molecular-weight organic acids can prominently enhance P release from the soil and improve P absorption by crops, thereby alleviating P stress [22]. This is exemplified by radish (*Raphanus sativus* L.) and oilseed rape (*Brassica napus* L.), in which P stress induces specific root exudation of organic acids to improve soil nutrient bioavailability and enable plant adaption to stress [23]. When a K-rich genotype of grain amaranth (*Amaranthus hypochondriacus* L.) is grown under a low K level, its root exudates contain abundant malic, citric, and oxalic acids, which can promote the release of soil mineral K [24]. Presently, the effects of Mg supply level on root growth and the relationship between nutrient absorption and root exudation of organic acids in different vegetable genotypes are still poorly understood.

Tomato (*Solanum lycopersicum* L.) and cucumber (*Cucumis sativus* L.) are typical species of solanaceous and gourd vegetables, respectively. Both crops are among the most widely cultivated and consumed vegetables worldwide, so their nutritional quality holds great value for human dietary nutrition and health. In the present study, tomato and cucumber were used to ascertain the effects of various Mg supply levels on root growth and nutrient absorption and accumulation in different genotypes of greenhouse vegetables. The aim of this study was to decipher the responses of tomato and cucumber to Mg deficiency and excess. The study’s results could provide empirical evidence for Mg fertilizer regulation in greenhouse vegetable production.

## 2. Results

### 2.1. Changes in Plant Biomass of Two Vegetable Crops under Different Mg Treatments

The total biomass per tomato plant and the biomass of various parts of tomato plants were significantly higher than those of cucumber plants when treated with the same concentrations of Mg (Table 1). Among the four treatments, the total biomass of tomato plants was 20.80–42.32% higher than that of cucumber plants. With increasing Mg concentration of nutrient solution, the biomass of both tomato and cucumber plants exhibited an upward trend until reaching the maximum values under the Mg2 treatment. The total biomass of tomato plants from the Mg2 treatment increased by 30.94%, 13.99%, and 13.99% compared with that of Mg0.2, Mg1, and Mg3 treatments, respectively. Greater increases were observed in cucumber plant biomass from the Mg2 treatment, by 54.27%, 17.40%, and 19.89%, respectively. Solution Mg concentration showed consistent effects on the shoot and total biomass of tomato and cucumber plants, but its effects on root biomass were different. Cucumber root biomass reached its peak value under the Mg2 treatment, but there was no significant difference in tomato root biomass among the Mg1, Mg2, and Mg3 treatments.

### 2.2. Responses of Root Growth in Two Vegetable Crops to Different Mg Supply Levels

**Figure 1:**
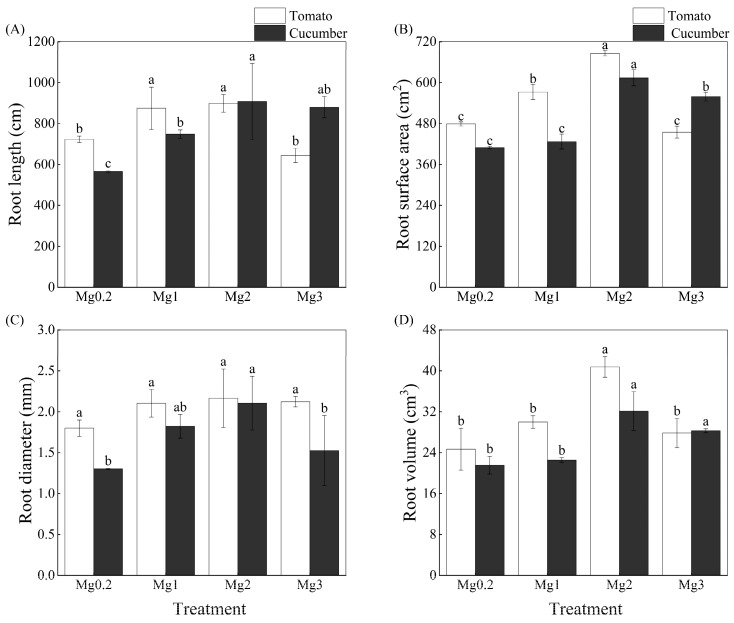
Effect of different Mg treatments on root length (**A**), root surface area (**B**), root diameter (**C**), and root volume (**D**) of tomato and cucumber plants. Four different levels of Mg were applied to both vegetable crops: 0.20 mmol/L (Mg0.2), 1.0 mmol/L (Mg1), 2.0 mmol/L (Mg2), and 3.0 mmol/L (Mg3). Root growth parameters were measured after 25 day of treatment. Error bars indicate standard deviation of the mean (*n* = 3). For each crop, different letters above the error bars indicate significant differences among various treatments at the 5% level.

All root growth parameters of tomato and cucumber plants trended high with increasing Mg concentration of the nutrient solution, and their maximum values were observed under the Mg2 treatment (Figure 1). This suggests that either too low or too high Mg supply level had an inhibitory effect on root growth in the different vegetable crops. Comparing the two crops revealed that the root surface area, root volume, and root diameter of tomato plants were larger than those of cucumber plants under the Mg0.2, Mg1, and Mg2 treatments. In contrast, cucumber plants showed a larger root length, root surface area, and root volume than tomato plants under the Mg3 treatment.

### 2.3. Differences in Nutrient Contents in Various Plant Parts of Two Vegetable Crops among Mg Treatments

**Figure 2:**
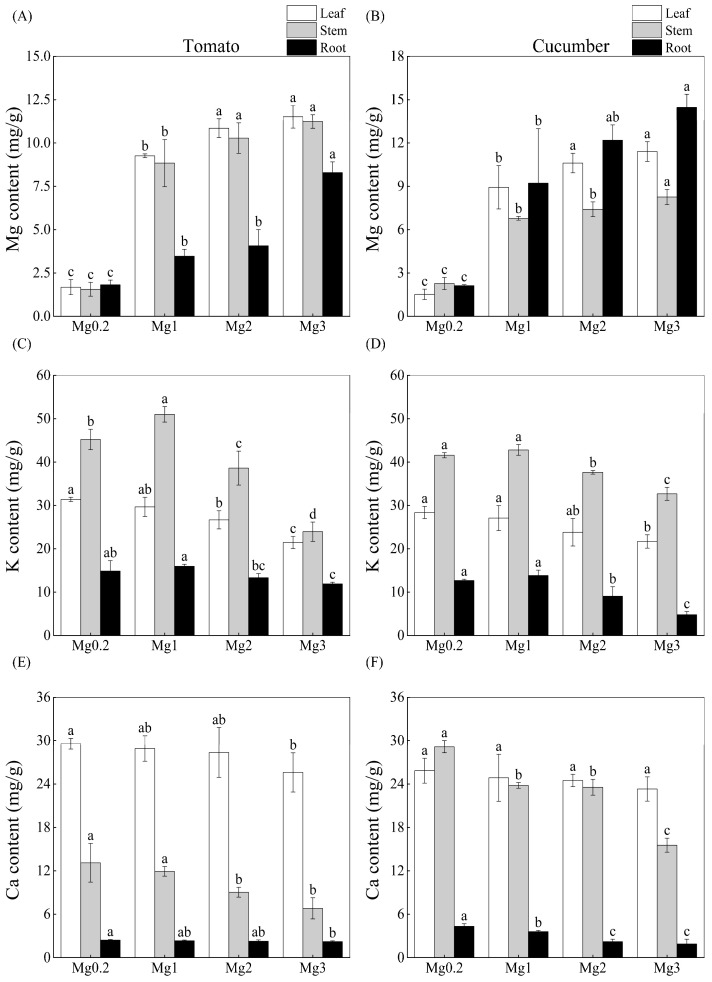
Effect of different Mg treatments on Mg contents (**A**,**B**), K contents (**C**,**D**), and Ca contents (**E**,**F**) in various parts of tomato and cucumber plants. Treatment abbreviations are defined in the caption of Figure 1. Nutrient contents were measured after 25 day of treatment. Error bars indicate standard deviation of the mean (*n* = 3). For each crop, different letters above the error bars indicate significant differences among various treatments at the 5% level.

The Mg contents in various parts of tomato and cucumber plants gradually increased with increasing Mg concentrations of the nutrient solution (Figure 2A,B). The shoot Mg content of tomato plants was higher than that of cucumber plants, whereas the opposite trend was observed for root Mg content across all treatments. The K contents in the leaves and roots of both crops significantly decreased with increasing Mg concentrations of the nutrient solution (Figure 2C,D). The K content of tomato shoots increased first and then decreased significantly with an increasing concentration of solution Mg, which contrasted with a continuous decrease in the K content of cucumber shoots. In most cases, the mean K contents of tomato leaves, shoots, and roots were higher than those of cucumber plant parts, except that the leaf and shoot K contents exhibited the opposite trend under the Mg3 treatment.

The Ca contents in various plant parts of both crops generally decreased with increasing concentrations of the Mg solution (Figure 2E,F). The decrease in the Ca content of tomato leaves was significant, whereas a non-significant decrease occurred in the Ca content of cucumber leaves. The mean Ca content in tomato leaves was higher than that of cucumber leaves across all treatments, in contrast to the pattern of shoot and root Ca contents in the two crops.

### 2.4. Patterns of Nutrient Accumulation in Two Vegetable Crops under Different Mg Treatments

**Figure 3:**
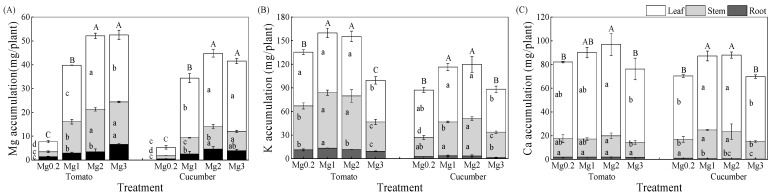
Effect of different Mg treatments on Mg accumulation (**A**), K accumulation (**B**), and Ca accumulation (**C**) in tomato and cucumber plants. Treatment abbreviations are defined in the caption of Figure 1. Values are the mean ± standard deviation (*n* = 3). Nutrient accumulation as measured after 25 day of treatment. Error bars indicate standard deviation of the mean (*n* = 3). For each crop, different lowercase letters indicate significant differences in the nutrient accumulation in individual plant parts among various treatments, and different uppercase letters indicate significant differences in total nutrient accumulation per plant among various treatments at the 5% level.

The Mg accumulation in various parts of tomato plants and their total Mg accumulation per plant continuously increased with increasing Mg concentration of the nutrient solution and peaked under the Mg3 treatment (Figure 3A). The total Mg accumulation in tomato plants from the Mg3 treatment was 6.85, 1.32, and 1.01 times higher compared with that of Mg0.2, Mg1, and Mg2 treatments, respectively. With increasing Mg concentration of the solution, the Mg accumulation in various parts of cucumber plants and their total Mg accumulation per plant also increased until reaching the maximum values under the Mg2 treatment. The total Mg accumulation in cucumber plants from the Mg2 treatment was 8.44, 1.30, and 1.08 times higher compared with that of Mg0.2, Mg1, and Mg3 treatments, respectively.

The K and Ca accumulation in various parts of tomato plants increased gradually with increasing Mg concentration of the solution from 0.2 to 1.0 mmol/L, which mirrored the pattern of total K and Ca accumulation per tomato plant (Figure 3B,C). A further increase in the Mg concentration of the solution from 2.0 to 3.0 mmol/L resulted in decreased K and Ca accumulation in tomato plants. Similar trends were observed in cucumber plants, although the total accumulation of both K and Ca per plant reached the peak values under the Mg2 treatment. The mean accumulation of K, Ca, and Mg in tomato plants exceeded that of cucumber plants across all treatments, indicating that tomato crops outperformed cucumber crops in nutrient accumulation.

### 2.5. Comparison of Root-Exuded Organic Acids in Two Vegetable Crops Supplied with Different Mg Levels

Solution Mg concentration exhibited distinct effects on the types and contents of organic acids exuded by the roots of tomato and cucumber plants (Table 2). Four organic acids were detected in tomato root exudates, whereas only three organic acids were found in cucumber root exudates. When the solution Mg concentration varied from 1.0 to 3.0 mmol/L, the para-hydroxybenzoic acid (PHBA) and oxalic acid (OA) contents in root exudates of both crops noticeably increased. Higher contents of para-hydroxybenzoic acid (PHBA) and oxalic acid (OA), as well as more types of phenolic acids (e.g., cinnamic acid), were observed in root exudates from the plants with the Mg0.2 treatment compared with those with the Mg1 and Mg2 treatments. A comparison between the two different crops revealed that benzoic acid (BA) was exuded by tomato roots but not by cucumber roots. The root exudation pattern of benzoic acid (BA) mirrored that of para-hydroxybenzoic acid (PHBA) and oxalic acid (OA) in tomato plants. With the exception of the Mg1 treatment, the mean para-hydroxybenzoic acid (PHBA) content in tomato root exudates from different treatments was higher than that of cucumber root exudates. The results indicate that a deficient supply of Mg (Mg0.2 treatment) promoted crop roots to exudate organic acids and increased the types of exuded organic acids, whereas an excessive supply of Mg enhanced root exudation of phenolic acids (PHBA, BA) in different vegetable crops.

## 3. Discussion

### 3.1. Effects of Mg Supply Level on Nutrient Absorption and Root Growth in Different Vegetable Genotypes

Mg deficiency has become a potential limiting factor for crop yield in agricultural production. In this study, Mg deficiency occurred in both vegetable crops (Mg content < 0.2%) under the low level of Mg supply (0.2 mmol/L), which strongly affected biomass formation in various plant parts and the total plant (Table 1). Increasing the Mg supply level caused no reduction in Mg absorption in tomato and cucumber crops under hydroponic conditions. In particular, Mg absorption was substantially enhanced in tomato and cucumber roots under increased Mg supply levels (Figure 1). Indeed, reasonable applications of Mg fertilizer can increase crop yield, improve fruit quality, and enhance plant resistance to adverse stresses [14,25].

Mg supply at the high Mg level (3.0 mmol/L) led to Mg overabsorption and inhibited K and Ca absorption in tomato and cucumber crops (Mg content > 0.2%; Figure 2). Therefore, the excessive application of Mg fertilizer would lead to the waste of Mg resources and cause Ca and K deficiencies in crop plants during practical production. The accumulation of K and Ca in various plant parts and their total accumulation per plant decreased in response to Mg excess (Figure 3). This indicates that the response patterns of K and Ca absorption to Mg excess were consistent in different vegetable genotypes, and both showed antagonistic effects. Moderately increasing the Mg supply level induced a synergistic effect between K and Mg in the shoots of tomato plants, whereas high Mg supply severely inhibited K absorption due to an antagonistic effect.

**Figure 4:**
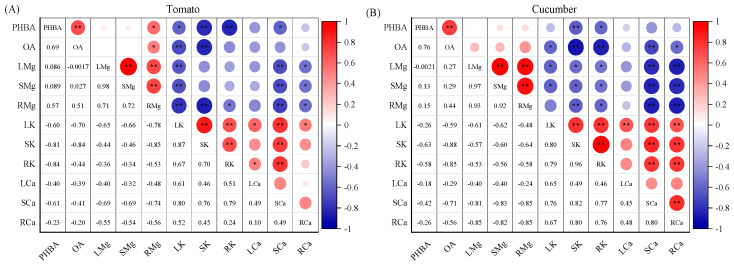
Correlation between nutrient absorption and root exudation of organic acids in tomato (**A**) and cucumber (**B**) plants under different Mg treatments. PHBA, para-hydroxybenzoic acid; OA, oxalic acid; LMg, SMg, and RMg represent leaf Mg, shoot Mg, and root Mg, respectively; LK, SK, and RK represent leaf K, shoot K, and root K, respectively; LCa, SCa, and RCa represent leaf Ca, shoot Ca, and root Ca, respectively. * *p* < 0.05 and ** *p* < 0.01. The size of the circle represents the size of the correlation coefficient.

The results of the correlation analysis provided further evidence for the antagonistic effect between Mg and K (or Ca) in crops. There was a negative correlation between K and Mg contents, as well as between Ca and Mg contents, in various plant parts of both tomato and cucumber crops. In contrast, a positive correlation emerged between K and Ca contents in various plant parts (Figure 4). Vegetable growers tend to apply large amounts of fertilizers, causing the accumulation of excessive salts (e.g., Mg^2+^, Ca^2+^, and Na^+^) in the soil [26]. The massive accumulation of salts would lead to elemental antagonism and thereby strongly inhibit the plant’s absorption of other nutrients [8,27]. It has been reported that increasing the supplied concentration of Mg^2+^ ions can reduce the relative quantity of Mg^2+^ ions adsorbed by xylem conduit walls and improve ion transport efficiency to the shoots. However, supplying Mg^2+^ ions at too high concentrations may cause antagonism between Mg^2+^ and K^+^ (or Ca^2+^) ions due to their competition for ion channels or the requirement of the maintenance of electroneutrality [28,29]. Therefore, Mg fertilizer should be applied rationally in agricultural production to prevent declines in plant yield and quality caused by ion antagonism.

This study showed that Mg absorption varied between the two vegetable genotypes, as indicated by the fact that Mg absorption under increased Mg supply was higher in shoots and leaves of tomato compared to cucumber, while cucumber roots had higher Mg absorption compared to tomato in all treatments (Figure 2A,B). Additionally, the two crops differed in root growth and nutrient absorption (K, Ca) under the experimental conditions. Irrespective of the Mg supply level, the total biomass per plant (Table 1) and root growth parameters (Figure 1) of tomato plants were higher than those of cucumber plants. Excluding Ca in the shoots and roots, the mean K and Ca contents in various parts of tomato plants were higher than those of cucumber plants (Figure 2). However, cucumber plants showed superior root growth (Figure 1) and K absorption (Figure 2C,D) characteristics compared with tomato plants under a high Mg supply. Collectively, these results indicate that various vegetable crops responded differently to Mg deficiency and excess.

This study indicated that tomato roots grew better than cucumber roots under a deficient or moderate supply of Mg, which facilitated nutrient absorption and contributed to the accumulation of dry matter and nutrients. In contrast with tomato plants, cucumber plants showed a certain tolerance to Mg excess. In addition to external environmental factors (e.g., Mg concentration, temperature, light), the genetic traits of plants (i.e., differences between cultivars) also had a great influence on Mg absorption. Remarkable differences have been found in the Mg absorption and translocation characteristics of various tomato cultivars [30]. Similar results are reported for other crops, such as Jerusalem artichoke (*Helianthus tuberosus* L.), sweet potato (*Ipomoea batatas* Lam.), and grape (*Vitis vinifera*) [31,32,33]. Given the different absorption characteristics of Mg in various crops and cultivars, attention should be paid not only to reasonable regulation of Mg fertilizer but also to distinct plant growth and nutrient-absorption characteristics in agricultural production.

### 3.2. Relationship between Crop Nutrition and Root Exudation of Organic Acids

In this study, a deficient supply of Mg led to increased contents of para-hydroxybenzoic acid (PHBA) and oxalic acid (OA) and more types of organic acids in root exudates from both tomato and cucumber crops (Table 2). This indicates that Mg deficiency induced crop roots to exude more organic acids in terms of quantity and composition, which could improve soil nutrient bioavailability and thereby alleviate nutrient stress [22]. An excessive supply of Mg caused the roots to exude phenolic acids (e.g., para-hydroxybenzoic acid; Table 2), which could disrupt cell membrane structures and prevent oxidative phosphorylation reactions in crop plants, adversely affecting nutrient absorption and use efficiency. A previous study has shown that in P-stressed crops, such as legumes, grasses, and crucifers, the amount of root-exuded organic acids increases considerably, mainly including citric acid, malic acid, oxalic acid, tartaric acid, succinic acid, and γ-aminobutyric acid [22].

Excessive levels of root exudates can pose autotoxic effects on plants, and phenolic acids are recognized as the most important allelochemicals in root exudates [34]. When accumulating in the soil, excessive phenolic acids can reduce the bioavailability of soil nutrients [35]. Phenolic acids also decouple oxidative phosphorylation [36], leading to oxidative stress in plants. This stress would impair cell membrane structure and function through lipid peroxidation, consequently affecting plant biological activities such as ion absorption, cell division, and photosynthesis [37,38,39]. Phenolic acids reportedly show inhibitory effects on vegetable crops, such as pakchoi (*Brassica chinensis* L.) and tomato [40,41]. Furthermore, benzoic acid (BA) was detected in tomato root exudates but not in cucumber root exudates (Table 2). The results demonstrate that root exudation of organic acids in various vegetable genotypes responded differentially to Mg supply level.

Based on the correlation analysis (Figure 4), the para-hydroxybenzoic acid (PHBA) content and oxalic acid (OA) content in root exudates was negatively correlated with the K and Ca contents in various parts of tomato plants (*p* < 0.05 or 0.01). The correlations between the para-hydroxybenzoic acid (PHBA) and oxalic acid (OA) contents in root exudates and the nutrient contents in various parts of cucumber plants were consistent with those observed in tomato plants. These results indicate that root-exuded phenolic acids adversely affected nutrient absorption in different vegetable genotypes under increased Mg supply levels. Accordingly, excessive application of Mg fertilizer in production may lead to massive exudation of phenolic acids by crop roots, which not only hinders their own nutrient uptake but also influences the surrounding crop plants.

This study provides additional evidence for the close association between nutrient absorption and root exudation of organic acids in different vegetable genotypes. Based on the results obtained under different Mg supply levels, this study shows that crop roots can effectively absorb Mg through root exudation of organic acids. Further study should be conducted to assess the mutual relationship between nutrient absorption (i.e., Mg, K, Ca) and root exudation of organic acids, which will provide guidance for efficient nutrient use in vegetable crops.

## 4. Materials and Methods

### 4.1. Experimental Materials

The experiment was conducted in a multi-span glass greenhouse at the Agricultural Science Training Base of Ningxia University (Yinchuan, China) from March through May 2023. The greenhouse was well designed for ventilation and light supplementation, as well as for temperature and humidity control. The mean temperature in the greenhouse was 24 ± 3 °C (day)/18 ± 3 °C (night), and the relative humidity was ~50%. The tomato cultivar used in the experiment was *S. lycopersicum* cv. “Saina”, with plants having infinite growth and mature fruits in a deep pink color with high glossiness. The cucumber cultivar used in the experiment was *C. sativus* cv. “Jianyan-1”, with plants having large plump leaves in a dark green color and three to five side vines per plant. Both tomato and cucumber seeds were purchased from Ningxia Tianyuan Seed Industry Co., Ltd. (Yinchuan, China). Seedlings were grown in 72-cell plug trays containing a mixture of peat soil, perlite, and vermiculite (1:1:1, *v*/*v*/*v*) and no fertilizer. Seedlings with uniform growth were selected for the hydroponic experiment when they had four fully developed true leaves and one bud.

### 4.2. Experimental Design and Treatments

The basal nutrient solution was prepared with pure water (containing 0.20 mmol/L Mg^2+^ and 0.17 mmol/L K^+^) based on the formula of Yamazaki’s nutrient solution for tomato [42]. Four different levels of Mg were applied to both vegetable crops: 0.20 mmol/L (Mg0.2), 1.0 mmol/L (Mg1), 2.0 mmol/L (Mg2), and 3.0 mmol/L (Mg3). The experiment used a one-way completely randomized design with four treatments and four replications. In addition to Mg^2+^ supplied by MgSO_4_ for different treatments, other ions were provided with the following reagents (mmol/L): Ca(NO_3_)_2_·4H_2_O (1.500), KNO_3_ (4.000), NH_4_H_2_PO_4_ (0.661), Na_2_Fe-EDTA (0.038), H_3_BO_3_ (0.019), MnCl_2_·4H_2_O (0.004), ZnSO_4_·7H_2_O (0.003), CuSO_4_·5H_2_O (0.002), and (NH_4_)_6_Mo_7_O_12_ (0.01 × 10^−3^). Seedlings were transplanted in pots with two holes (inner diameter: 6.0 cm) and placed in a 15 L hydroponic tank. There were two seedlings per hole, and a total of 16 pots were prepared for the two crops. After 5 day of seedling recovery with pure water, 13 L of 1/4 Yamazaki’s nutrient solution was added to the hydroponic tank for 10 day of treatment, followed by an equal volume of 1/2, 3/4, and full nutrient solutions for 5 day each. Therefore, a total of 25 day of nutrient solution treatment was used in this experiment. The hydroponic tank was connected to an oxygenation device that supplied oxygen (40 L/min) for 30 min at 2 h intervals. The solution pH was controlled in the range of 5.5–6.5, and the nutrient solution was replaced weekly.

### 4.3. Sampling and Testing

#### 4.3.1. Root Exudate Collection and Analysis

**Figure 5:**
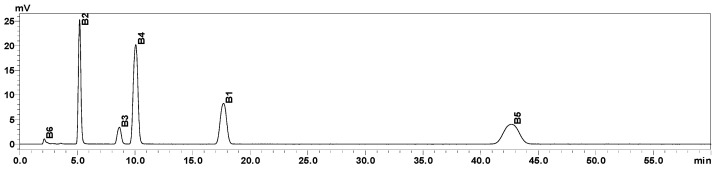
Chromatogram of six organic acid standards: B1–B6 are benzoic acid, para-hydroxybenzoic acid, para-coumaric acid, ferulic acid, cinnamic acid, and oxalic acid, respectively.

Root exudates were randomly collected from plants in three holes per treatment after 25 day (flowering period) of incubation under different Mg levels. Whole plants were removed carefully from the hydroponic tank to avoid damage to the roots. After repeated washes with distilled water, the roots were placed in 250 mL plastic bottles containing 200 mL of distilled water and incubated at 20 ± 2 °C for 24 h. After that, the roots were cut off and collected, with the water extract filtered through a 0.45 μm microporous filter membrane (Jinlong Co., Ltd., Tianjin, China). The filtrate was concentrated to 20 mL in a 40 °C water bath using a rotary evaporator (Yarong Biochemical Instrument Factory, Shanghai, China) and then stored at −20 °C until use. Organic acid standards (purity > 98%) were purchased from Solarbio Science & Technology Co., Ltd. (Beijing, China). An ultra-fast liquid chromatograph (UFLC; Shimadzu, Kyoto, Japan) equipped with an ultraviolet detector was used to determine organic acid contents in root exudates. The UFLC analysis was carried out on a C18 column (150 mm × 4.6 mm, 5 μm; Hitachi, Tokyo, Japan). The mobile phase consisted of solution A (0.05 mol/L KH_2_PO_4_, pH 2.5) and solution B (methanol: acetonitrile = 60:40, *v*/*v*) at a 25:75 ratio (*v*/*v*). Other chromatographic conditions were as follows: column temperature, 35 °C; flow rate, 1.0 mL/min; sample size, 10 μL; and detector wavelength, 245 nm. The chromatogram of six organic acid standards is shown in Figure 5.

#### 4.3.2. Plant Growth and Nutrient Analysis

After the collection of root exudates, root growth parameters were analyzed using a root scanner (Epson, Seiko Epson Corp., Nagano, Japan) and a root analysis system (WinRHIZO, Rengent Instruments Inc., Quebec, Canada). Briefly, a small amount of water was added to the sample tray on the root analysis system, and the root sample was spread in the water on the tray. The roots were scanned to acquire images, and total root length, root surface area, root volume, and mean root diameter were measured.

Whole plants were divided into root, shoot, and leaf samples. Each part was deactivated in an oven at 105 °C for 30 min and then dried at 70 °C until constant weight. After the dry weight of various plant parts was recorded, the dry samples were crushed and passed through a 0.18 mm sieve. A 0.5 g sample was then weighed into a crucible and completely carbonized at a low temperature (200 °C) in an electric furnace. Afterward, the crucible was placed in a muffle furnace for ashing at 550 °C for 6 h. The ash sample was dissolved in mixture of distilled water and nitric acid (1:1, *v*/*v*) and then diluted to constant volume, with LaCl_3_ added as a masking agent. A standard stock solution containing 10 μg/mL of Mg was used to formulate standard working solutions at the concentrations of 0, 0.2, 0.4, 0.8, 1.2, and 2.0 μg/mL. A standard stock solution containing 100 μg/mL of Ca was used to formulate standard working solutions at the concentrations of 0, 2.0, 4.0, 8.0, 12, and 20.0 μg/mL. The Mg and Ca concentrations in acid extracts were measured using a Z-2000; an atomic absorption spectrophotometer (Hitachi) with a combustor height of 7.5 mm, and a wavelength of 285.2 nm. A standard stock solution containing 100 μg/mL of K was used to formulate standard working solutions at different concentrations (0, 10, 20, 30, 40, 50 μg/mL), and K analysis was conducted using a FP640 flame photometer (Yidian Analytical Instrument Co., Shanghai, China). The concentrations of Mg, K, and Ca in the samples were calculated by preparing standard curves.

### 4.4. Data Analysis

Data processing and calculation were executed using Excel v2021 (Microsoft Corp., Redmond, WA, USA). For plant growth parameters, nutrient contents, and root-exuded organic acid contents, one-way analysis of variance with Duncan’s test was conducted at the 5% level using SPSS v25.0 Statistics (IBM Corp., Armonk, NY, USA). Pearson correlation analysis was carried out between the K, Ca, and Mg contents in various plant parts and the para-hydroxybenzoic acid (PHBA) and oxalic acid (OA) contents in root exudates. All plots were created using Origin v2021 (OriginLab Corp., Northampton, MA, USA).

## 5. Conclusions

This study deciphered the response patterns of tomato and cucumber crops to Mg deficiency and excess under hydroponic conditions. When the Mg supply level was moderately increased from 0.2 to 2.0 mmol/L, plant biomass, root growth, nutrient accumulation, and root exudation of organic acids all improved in different vegetable crops, especially in tomato plants. An excessive increase in Mg supply level to 3.0 mmol/L resulted in inhibitory effects on both crops, and cucumber plants showed a certain tolerance to high Mg supply. Increasing the Mg supply level promoted Mg absorption and accumulation while hindering K and Ca absorption in the two crops. A deficient supply of Mg promoted root exudation of organic acids and increased the types of exuded organic acids, which could facilitate plant adaptation to nutrient stress. An excessive supply of Mg led to massive exudation of phenolic acid by the roots, negatively affecting nutrient absorption. In addition to the rational application of fertilizer resources, different nutrient supplies should be appropriately matched for various crops in order to improve their yield and nutritional quality in greenhouse cultivation. In this experiment, a nutrient solution was used as the cultivation medium, and the physicochemical properties were different from those of the soil. Moreover, soil cultivation in actual facilities may be affected by a variety of disturbing factors, such as salt leaching and microbial activities. Therefore, the next study needs to further explore the effects of different magnesium supply levels on nutrient absorption and the organic acid secretion of vegetables cultivated in facilities’ soil.

## Figures and Tables

**Table 1:** Effect of different Mg treatments on plant biomass of two different vegetable crops grown in a greenhouse (g/plant dry weight). Four different levels of Mg were applied to both vegetable crops: 0.20 mmol/L (Mg0.2), 1.0 mmol/L (Mg1), 2.0 mmol/L (Mg2), and 3.0 mmol/L (Mg3). Plant biomass was measured after 25 day of treatment. Values are the mean ± standard deviation (*n* = 3). Different lowercase letters in the same column indicate a significant difference among various treatments at the 5% level.

Treatment	Tomato			Cucumber		
	Shoot Biomass	Root Biomass	Total Biomass	Shoot Biomass	Root Biomass	Total Biomass
Mg0.2	3.41 ± 0.13 c	0.76 ± 0.04 b	4.17 ± 0.17 c	2.70 ± 0.06 c	0.22 ± 0.01 b	2.93 ± 0.06 c
Mg1	3.95 ± 0.04 b	0.84 ± 0.03 ab	4.79 ± 0.07 b	3.59 ± 0.11 b	0.26 ± 0.03 b	3.85 ± 0.07 b
Mg2	4.59 ± 0.02 a	0.87 ± 0.07 a	5.46 ± 0.09 a	4.15 ± 0.15 a	0.37 ± 0.05 a	4.52 ± 0.20 a
Mg3	4.00 ± 0.11 b	0.79 ± 0.01 ab	4.79 ± 0.10 b	3.50 ± 0.01 b	0.27 ± 0.06 b	3.77 ± 0.04 b

**Table 2:** Effect of different Mg treatments on organic acid contents in root exudates of tomato and cucumber plants (mg/L). Treatment abbreviations are defined in the footnote of Table 1. Values are the mean ± standard deviation (*n* = 3). BA, benzoic acid; PHBA, para-hydroxybenzoic acid; PCA, para-coumaric acid; FA, ferulic acid; CA, cinnamic acid; and OA, oxalic acid. Root exudation of organic acids was analyzed after 25 day of treatment. Different lowercase letters in the same column indicate significant differences among various treatments at the 5% level.

Treatment	Tomato						Cucumber					
	BA	PHBA	PCA	FA	CA	OA	BA	PHBA	PCA	FA	CA	OA
Mg0.2	1.94 b	1.73 b	/	/	1.71	346.29 b	/	1.69 b	/	/	1.39	330.37 a
Mg1	0.79 d	1.41 c	/	/	/	219.00 d	/	1.50 d	/	/	/	236.40 c
Mg2	1.32 c	1.71 b	/	/	/	243.74 c	/	1.55 c	/	/	/	282.86 b
Mg3	2.78 a	2.94 a	/	/	/	389.09 a	/	2.85 a	/	/	/	326.20 a

## Data Availability

Data are contained within the article.
